# Effects of daylength manipulation on migratory activity and fuelling in a long-distance nocturnal songbird migrant

**DOI:** 10.1007/s00359-025-01772-3

**Published:** 2025-10-25

**Authors:** Susanne Åkesson, Mihaela Ilieva, Giuseppe Bianco

**Affiliations:** 1 Department of Biology, Lund University, Ecology Building, 223 62 Lund, SE Sweden; 2 Institute of Biodiversity and Ecosystem Research, Bulgarian Academy of Sciences, 2 Gagarin Str., 1113 Sofia, Bulgaria

**Keywords:** Bird migration, Endogenous migration program, Eurasian reed warbler, Migratory restlessness, Photoperiod

## Abstract

Migratory birds have evolved a multitude of physiological and behavioural adaptations to reach their population-specific wintering areas during their first migration. The endogenous program encodes distance, direction and fuelling, and involves species-specific adaptations leading naïve migratory birds along highly diverse routes. While daylength has been extensively studied in relation to the onset of migration, its potential role in the transition out of the migratory phenotype remains largely untested. Here we study, by experimentally increasing the daylength in autumn simulating a temporal shift earlier in the season or a latitudinal displacement toward the wintering area as experienced later in the season after the autumn equinox, what effect a substantial photic treatment has on overall level and diel pattern of activity and fuelling in juvenile Eurasian reed warblers (*Acrocephalus scirpaceus*) migrating to tropical Africa. The treatment group experienced a 2h increase in daylength in the evening, while the control group was held in the local photoperiod in southern Sweden. The controls showed strictly nocturnal migratory restlessness starting immediately after sunset, while the treatment birds responded by delaying the onset of nocturnal migratory restlessness following the artificially delayed sunset, without changing the level of activity. Treatment birds increased fuelling initially, but then reduced it after one week in captivity, resulting in a lower fuelling rate as compared to the controls by the end of experiment. The reduced fuelling suggests treatment birds possibly interpreted the diel period as arrival to the wintering area. The results confirm the importance of photic information in regulating phenotypic expressions of migratory activity and fuelling in juvenile birds.

**Supplementary Information:**

The online version contains supplementary material available at 10.1007/s00359-025-01772-3.

## Introduction

Migratory birds express a multitude of physiological, morphological, and behavioural adaptations (Åkesson and Hedenström 2007), enabling them to follow diverse routes and to cope with long-distance migrations. The unique migratory phenotypes typical for birds are shaped by natural selection, promoting range expansion to new breeding areas, following the outline of continents, and sometimes including barrier crossings (Alerstam et al. 2003). The endogenous migration program, guiding solo-migrating birds on their first migration is strictly inherited and encodes time, distance, direction and fuelling events (Berthold 1996; Helbig 1996; Gwinner 1996; Pulido 2007; Liedvogel et al. 2011). The functional characteristics of the program enable flexibility in migratory adaptations and promote diversification (Åkesson and Helm 2020), but still there are aspects of how the genetic program interacts with external information that are not completely understood and need to be further investigated. This refers especially to how the endogenous program and expression of migration phenotypic components in different bird species are affected by geomagnetic information and the diel cycle, that may vary with latitude (e.g. Fransson et al. 2001; Henshaw et al. 2009; Boström et al. 2010, 2012a; Ilieva et al. 2018; Müller et al. 2018; Åkesson et al. 2021).

Migration has evolved repeatedly in birds (Helbig 2003), and their long-distance movements are guided by different sources of geophysical information for orientation (e.g. Able 1980; Åkesson et al. 2014, 2017). How birds navigate is, however, much less understood. It has been suggested that geomagnetic information can provide latitudinal information for navigation (Fischer et al. 2003), but geomagnetic parameters can also form bi-coordinate maps (Wallraff 1990) that can be used by birds on regional or even global scales (Åkesson 1996; Phillips 1996; Åkesson and Alerstam 1998; Boström et al. 2012b). Geomagnetic information can affect fuelling decisions in migratory birds, leading to extensive fuelling in front of a barrier when the anticipated magnetic field parameters were experimentally exposed to the birds (Fransson et al. 2001; Ilieva et al. 2023), but also reduction in fuelling as birds are exposed to the geomagnetic parameters anticipated at their wintering areas (Kullberg et al. 2007; cf. Ilieva et al. 2016, 2018). Magnetic information has, furthermore, been shown to regulate migratory activity, resulting in reduced activity when the birds were exposed to the expected magnetic field at the wintering areas (Ilieva et al. 2018), adjustment of their remaining nocturnal activity along the magnetically simulated journey (Bulte et al. 2017), and modified endocrine levels to promote behavioural flexibility adapted to migration (Henshaw et al. 2009).

Photoperiod is a source of geophysical information that vary extensively with latitude and time of year and, thus, may pose challenges to avian migrants on long-distance migrations (Åkesson et al. 2017). The diel period can be used to define latitude and longitude on migrations and can regulate diurnal and seasonal rhythms (Aschoff 1960) resulting in species-specific variations that can be explained by ecological situations under which the timekeeping evolved in different species of birds (Helm et al. 2017). The diel cycle is an important trigger for migratory departures in birds following a circannual program (Gwinner 1996; Gwinner and Helm 2003; Helm et al. 2009; Helm and Liedvogel 2024). An experimentally increased diel cycle (day length) in the morning has recently been shown to affect activity and associated fuelling decisions in two songbird migrants, with the strongest effect observed for diurnally migrating dunnocks (*Prunella modularis*) as compared to nocturnally migrating European robins (*Erithacus rubecula*) (Åkesson et al. 2021).

Nocturnal bird migrants express migratory activity at night, so-called “Zugunruhe” or migratory restlessness (Wagner 1930), during the migration seasons. Both timing and length of the migratory restlessness periods have been shown to be correlated with anticipated migratory distance in songbirds (Berthold 1973; Maggini and Bairlein 2010). The endogenous migration program with its inherited variation between species, populations and individuals combined including adaptive responses to cope with varying environmental conditions along the route leads to the high flexibility in migratory performance observed in birds (Åkesson and Helm 2020). The migratory restlessness in birds is expressed at specific time of day and night for diurnal and nocturnal bird migrants, respectively, and for periods of different length of the migration season for intermediate (temperate zone) and long distance (tropical) migrants (e.g. Ilieva et al. 2018, 2023; Åkesson et al. 2021; Huffeldt et al. 2024). The diel activity pattern and seasonal migratory period are controlled by predictable photic information as a result of geophysical cycles extending over a day or a full year (Aschoff 1960; Gwinner 1996; Gwinner and Brandstatter 2001). An endogenous oscillator (i.e. circadian pacemaker) controls the annual migratory restlessness, which in absence of seasonal diel variations starts to free-run as shown for songbirds kept in cages for several years (Gwinner 1986). The diel oscillator is also dependent on control mechanisms from the circadian photoperiodic cycle, including variations of light levels (e.g. Aschoff 1960; Daan 1976; Gwinner 1996), but the oscillator can remain functional for several days in dim light (Coppack et al. 2008). The complex circadian pace making system in birds include three main components (i.e. encephalic photoreceptors, pineal and retina) and enables coordination of time over annual and circadian cycles, as well as during long-distance migrations (Gwinner and Brandstätter 2001). What effect the circadian pacemaker, including photoperiodic control, has on different functional components of the endogenous migration program, and in particular fuelling decisions in songbirds has received limited attention (cf. Müller et al. 2018; Åkesson et al. 2021; Huffeldt et al. 2024).

Here we study how an experimentally altered diel period affects timing and level of migratory restlessness and fuelling in a nocturnal passerine migrant, the Eurasian reed warbler (*Acrocephalus scirpaceaus*), wintering in tropical Africa. We investigated the expression of migratory restlessness throughout the day and night cycle for juvenile reed warblers captured on autumn migration in southern Sweden. The birds were kept in captivity for 14 days during which time we measured mass increase, food consumption and the birds’ activity in circular cages starting from the second day in captivity lasting 13 days. The control birds were kept in the local photoperiod, while the treatment birds experienced a prolonged day by 2h. We hypothesised that photoperiod would be a strong trigger of timing of activity with potential effects also on fuelling as the cue can provide both temporal and spatial information (Åkesson and Helm 2020). More specifically, we expected the birds’ activity rhythm to be affected by the increased photoperiod in the end of the day, such that the treatment birds would initiate nocturnal restlessness 2h later than the control birds following the experienced time of sunset. We predicted that the nocturnal restlessness either would extend over the complete night period and end at sunrise or follow a predisposed similar length of time for migratory restlessness as recorded for the control birds, unique to reed warblers that are long-distance migrants and has evolved to cover a species-specific distance between the breeding and wintering areas (Berthold 1973). During daytime, we expected birds to express lower levels of movement associated with foraging in the cages (Ilieva et al. 2018). We further predicted that the treatment birds given access to a 2h prolonged diel period would increase their fuelling rate (Kvist and Lindström 2000), and as a result of increased fuelling would put on larger fat deposits as compared to the controls. By keeping the geomagnetic cues at local values, we expected that any behavioural or physiological responses should be expressed in relation to the photic manipulations.

## Material and methods

We selected to work with Eurasian reed warblers that breed in reedbeds near the shores of freshwater lakes and rivers in Europe and are nocturnal passerine long-distance migrants wintering in sub-Saharan Africa (Zink 1975). Reed warblers breeding in Sweden are known to migrate via Western Europe and the Iberian Peninsula to wintering sites located in western sub-Saharan Africa (Fransson and Hall-Karlsson 2009). Their main migration passage in southern Sweden is in August and September (Fransson and Hall-Karlsson 2008).

We captured juvenile migratory reed warblers in a reedbed area located in the vicinity (< 1km) of the Stensoffa Ecological Field Station in southwest Sweden (55°41′N 13°26′E) in the period 4–9 September 2019. Thereafter we monitored the migratory phenotype of individual birds for a total of 13 days, starting at 11 September, in a facility dedicated to record migratory restlessness and fuelling (Ilieva et al. 2016). We accommodated 4 groups of 4 randomly chosen individuals in 4 wooden houses (n = 16 birds in total). In all houses, the birds were exposed to the natural light–dark cycle through the semi-transparent roofs (Åkesson et al. 2021). In addition to the natural light, all houses were equipped with LED light source (Lumak Pro) with daylight colour temperature (6500 K) automatically controlled by an electronic timer (Åkesson et al. 2021). The LED lights were switched on at 17:00 in all houses when no contribution to the house luminosity was measurable (Åkesson et al. 2021) and switched off at 19:30 (local sunset time) in the 2 control houses and 2h later in the treatment houses (21:30). Hence, the treatment group was exposed to an artificial 2h delayed sunset, corresponding to 2h increased daylength asymmetrically to midday. We used an electronic light meter (TES 1330A) to measure light intensity provided by the lamps when it was complete dark outside, resulting in light levels comparable with the light intensity measured at sunrise and sunset in the houses (Åkesson et al. 2021).

**Fig. 1:**
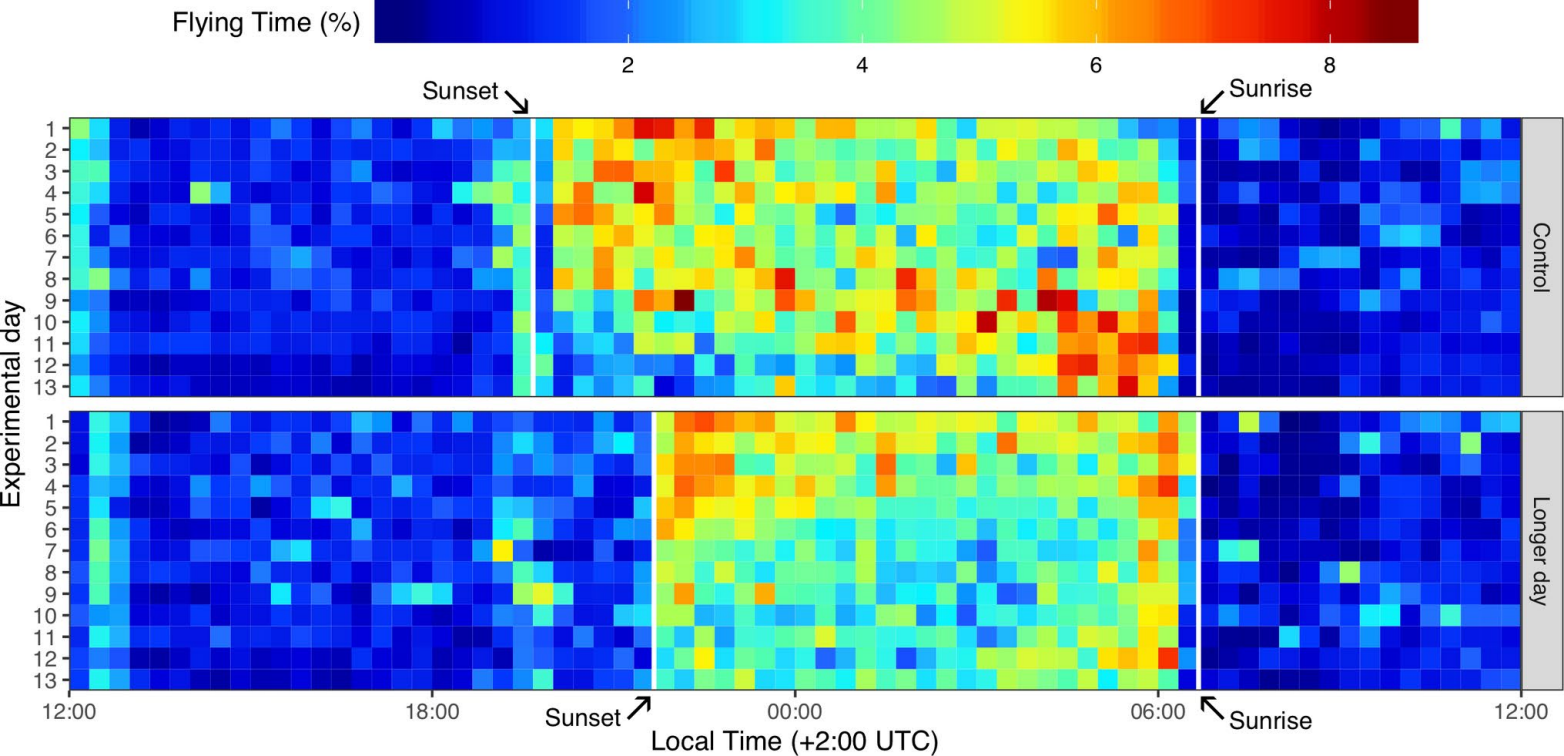
Actogram of first-year migratory reed warblers (*Acrocephalus scirpaceus*) monitored under local daylength timing (Control) and delayed sunset (Longer day). Each color-coded horizontal line shows the mean time spent in flying mode by the birds (n = 8 for each group) from 12:00 of each experimental day to 12:00 of the successive day. Solid white vertical lines indicate the time of sunset (19:30 for control) and 2h later (21:30 for longer day group) and sunrise at local time (06:30 for all groups)

**Fig. 2:**
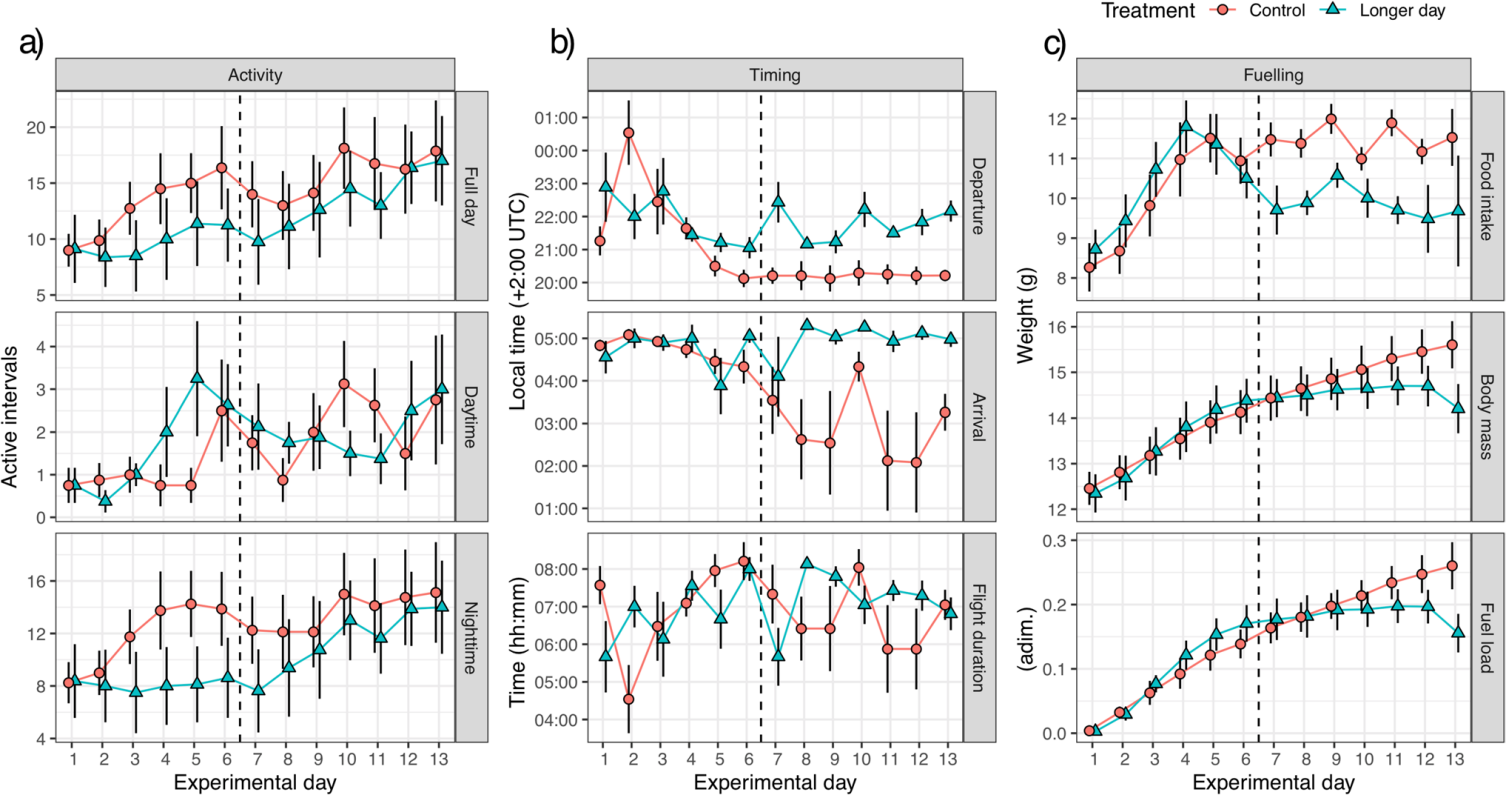
Migratory phenotype of first-year migratory reed warblers (*Acrocephalus scirpaceus*) monitored under local daylength timing (control) and delayed sunset (longer day) conditions (n = 8 for each group) measured as average (± SE) of: a) number of active intervals presented for the full day and divided in daytime and night-time periods; b) predicted time of departure and arrival and estimated flight duration; c) food intake, body mass and fuel load. Dashed vertical line separate the time period after which differences were found

All birds were held in individual non-magnetic circular cages (550 mm diameter by 700 mm height) and their behaviour was continuously monitored during the study period for four cages simultaneously by a network camera installed in the roof of each house (Ilieva et al. 2018; Åkesson et al. 2021). The films were thereafter analysed with a custom-made computer vision algorithm that detects when the bird is flying, that is, it is on its wings, excluding any other movement like walking, jumping, fluttering, etc. (Ilieva et al. 2018). This procedure was completely automated excluding potential observer-bias. We used flight as an indicator of migratory activity in our circular cages, where the birds could land on the side of the walls as well as on the centrally located circular perch. The flying time ratio measured for each bird in 20-min intervals (raw data available as Supplemental Material Appendix A1), was first summarised and visually inspected in the actogram in Fig. 1 and then used to extract detailed behavioural features as follows. We first measured the daily activity as the number of active intervals (i.e. when the bird was flying for more than 1 min in the 20-min interval; Ilieva et al. 2018) for the full experimental day (from 12:00 to 12:00 of the next day, local time), and thereafter the data was divided into a daytime period (from sunrise to sunset: 06:30—19:30 local time) and a night-time period (from sunset to sunrise: 19:30—06:30) (Fig. 2a). We then estimated the departure (i.e. initiation of the nocturnal restlessness period) and arrival time (i.e. end of nocturnal restlessness period) as the time at which birds that were active for at least 3 intervals (i.e., 1h) reached the 10th and the 90th percentile of the cumulative night-time activity, respectively (Schmaljohann et al. 2015; Åkesson et al. 2021; Fig. 2b). We finally calculated the daily duration of migratory activity for each bird as the time between the departure time and the arrival time as defined above (Fig. 2b).

The reed warblers were given food and water at a specific time every day (12:00–13:00, local time). At the same time, we measured the daily food consumed (remaining amount of food was subtracted from the amount of food given 24 h earlier) to the nearest 0.01 g and the body mass to the nearest 0.1 g of single individuals as outlined in Ilieva et al. (2016) and Åkesson et al. (2021) (Fig. 2c). The fuel load was calculated as the fraction of body mass increase relative to individual’s lean (fat-free) body mass (Åkesson et al. 2021; Fig. 2c).

For our statistical analysis, we used the software R version 3.6.1 (R Core Team 2019) and the *lme4* package version 1.1–21 (Bates et al. 2015). Activity interval counts were modelled against a Poisson distribution with a series of generalized linear mixed models; whereas timing of migration (departure time, arrival time, flight duration) and fuelling data (food intake and fuel load) were modelled with linear mixed effect models. All models had experimental day, treatment and their interactions as fixed effects and bird ID as random effect. We evaluated the contributions of the fixed factors comparing the models above against a depleted model missing the specific fixed effect and/or its interactions with the likelihood ratio test. Models were evaluated either for the entire duration of the experiment (days 1–13) or for a subset of the duration of the experiment (either days 1–6 or days 7–13). One-way ANOVA was used for initial body mass comparison.

## Results

The actogram of the control group showed a typical nocturnal behaviour in reed warblers with migratory restlessness expressed strictly during dark hours (i.e., between sunset and sunrise; Fig. 1). Control birds had a peak activity just before sunset, and after a short (20—30 min) quiescent period, they became very active and continued their intense activity until 20 min before sunrise (Fig. 1). Birds in the treatment group, on the other hand, awaited the time of 2h delayed sunset when the artificial lights went off to initiate their nocturnal migratory restlessness (Fig. 1). The time for the quiescent period in the treatment group expressed between the sunset and the start of the migratory activity, was not as clearly detectable in the actogram as for the control group. That is, reed warblers in the treatment group became active as soon as the artificial lights went off (Fig. 1). However, also the birds in the treatment group stopped the intense activity period just before the sunrise (Fig. 1). For both treatment groups we found an increased level of activity around 12:00 (local time), associated with the presence of the operator feeding the birds in the cages. However, this daily activity occurred several hours before any migratory behaviour was registered in this study (Fig. 1) and likely did not affect the activity expressed by the birds in the cages.

The migratory phenotype of reed warbles quantified in this study in terms of diel pattern of activity, timing of migration and fuelling strategy showed limited differences in the first part of the experiment (days 1—6), and substantial differences between the control and the treatment groups found in the last part of the experiment (days 7–13; Fig. 2). In fact, in the first part of the experiment, we only found the interaction between treatment and experimental day during night-time to be significant (days 1—6: χ^2^(1) = 7.2626, p = 0.007) when the control group was increasing the level of activity at higher rate as compared to the treatment group (Fig. 2a), for which the amount of night-time activity remained stable and low throughout the first period.

During the first 2–3 days of the experiment, both treatment groups showed a late departure after sunset (Fig. 2b), but at the same time, both groups had a consistent time of arrival (end of activity period) just before sunrise (Fig. 2b) with no difference found in departure or arrival time during the first part of the experiment (days 1–6). However, for the second part of the experiment (days 7–13), we found a significant effect of the treatment for both departure time (χ^2^(1) = 11.777, p < 0.001) and arrival time (χ^2^(1) = 6.8006, p < 0.001), but no effect of interaction between treatment and experimental day for both departure and arrival time. In other words, during the first part of the experiment, birds in both groups were keeping the same daily departure and arrival times (Fig. 1b); whereas, in the second part birds in both groups kept departure and arrival times constant but at different time of the day for the control (days 7–13, grand mean ± SD, departure: 20:23 ± 00:03; arrival: 03:06 ± 00:49) and the treatment group (departure: 21:58 ± 00:30; 05:08 ± 00:24) with a clear effect of the treatment with the treatment group departing later (i.e. initiating migratory restlessness) than the control group according with the delayed sunset and arriving (ending migratory activity) later than the control group (Fig. 2b).

We could not find any difference in duration of flight activity (Fig. 2b), meaning that, although both treatment groups varied their departing time during the experimental period (i.e. starting at a specific time relative to their respective experienced sunset time), both groups adjusted their arrival time to keep the flight duration constant throughout the experiment (Fig. 2b). This means that while departure, and indirectly arrival time, was affected by our treatment (external control), the flight duration was not affected, with the treatment group departing later due to the delayed sunset but stretching the flight duration further postponing the arrival time with a flight duration of approximately 7 h per night for both groups (Fig. 2b).

Both treatment groups showed the same foraging pattern with food intake increasing by 50% in the first part of the experiment (days 1–6) with no difference between control and treatment (Fig. 2c). In the second part of the experiment the food intake was constant within each group (no effect of interaction between treatment and experimental day), but food intake was at a different level between groups (significant treatment effect, days 7—13: χ^2^(1) = 10.098, p = 0.001) with the treatment group eating on average 14% less food as compared to the control group (Fig. 2c). The initial body mass of the two treatment groups was not different (control: mean ± SD: 12.5 ± 1.0 g; longer day: 12.3 ± 1.2 g; one-way ANOVA: F_1,14_ = 0.040, p = 0.844). However, the effect of lower food intake in the second part of the experiment affected the treatment group with a significant interaction of treatment and experimental day found (days 7—13: χ^2^(1) = 30.974, p < 0.001) with the consequences of a 9% lighter body mass for the treatment group (14.2 ± 1.5 g) as compared to the control group (15.6 ± 1.5 g) at the end of the experimental period (Fig. 2c), despite treatment birds having access to longer periods of daylight for foraging.

## Discussion

The delay of sunset time resulted in delayed initiation of nocturnal migratory activity in our treatment reed warblers but did not reduce the overall activity. Still, we found the level of activity was somewhat reduced for the treatment birds in the first half of the experimental period as compared to the second period. We also found the extent of the diel period used for nocturnal migratory activity throughout the experimental period was somewhat variable between days, but then similar between the treatment and control group. In the first half of the experiment, reed warblers of both groups seemed to have a quite variable, but relatively late, departure timing. Later in the second half of the experiment, both groups kept their departure at a constant time relative to the experienced sunset. Both groups kept a constant duration of migratory activity by adjusting the arrival (stop) time relative to their respective departure time. This supports the finding that the migratory departure time in reed warblers is adjusted relative to the light level and is triggered by an external photic signal, whereas the duration of migratory activity is instead controlled by an internal endogenous mechanism, in line with what has been found for nocturnal European robins and diurnally migrating Dunnocks (*Prunella modularis*) (Åkesson et al. 2021), unaffected by photic treatment. The length of the activity period and fuelling have been shown to be population-specific and connected to the anticipated migration distance (Berthold 1973; Maggini and Bairlein 2010), with recent findings supporting species-specific patterns associated with different migration strategies (Ilieva et al. 2023).

Our 2h-delay of sunset affected the fuelling pattern of the treatment reed warblers. Differences in the fuelling and timing of migration were found in the second part of the experimental period meaning that several physiological changes must have been initiated during the first week of the experiment, including e.g. synchronising of the internal clock with the new altered photoperiod, and building up fat storage to recover from fuel lost during migratory stints before capture (Ilieva et al. 2016), but the effect of those became obvious only after some time in captivity. Temporary modulations of multiple physiological mechanisms have been shown to be associated with the expression of the migration phenotype (Sharma et al. 2021). Physiological processes associated with fuelling at stopover takes a substantial amount of time during migration (Hedenström and Alerstam 1998) and is dependent on availability of food and time for foraging (Lindström 2003), but can also be affected by perceived predator presence (Bianco et al. 2025). In our experiments, the birds were provided food and water ad libitum and therefore the changes in fuelling rate in the treatment birds must have been affected by the manipulation of external information, i.e. the diel cycle and daylength. Energy expenditure and level of activity further contribute to the realized fuelling rate in migratory birds (Ilieva et al. 2016, 2018) and may be part of the explanation why we observed late departure timing in the first part of the experiment. In this situation, our reed warblers probably were trying to quickly build up fat reserves by reducing their flight activity by initiating nocturnal activity later in the night and reducing activity. This activity pattern in the first part of the experimental period was associated with birds quickly increasing the rate of food intake which also resulted in a substantial mass increase. The fuelling pattern may be explained by an inherited flexibility of the endogenous program regulated by fat level (Biebach 1985; Gwinner et al. 1985). A 2h prolonged day will provide longer time for foraging (Lindström 2003), which in turn could be used by the treatment birds to fuel faster during the initial period of our study. Contrary to this prediction, the treatment group exposed to a longer day was eating significantly less food during the second part of the study, which resulted in reduced body mass and fuel load toward the end of the experiment as compared to the control birds. We interpret the results such as the 2h longer day was perceived by the reed warblers as reaching the end of migratory season later in the autumn and arrival to their wintering area in tropical Africa (Zink 1975; Fransson and Hall-Karlsson 2008), as have recently been found in diurnally migrating dunnocks (*Prunella modularis*) (Huffeldt et al. 2024), and not a seasonal shift back in time. The migratory activity, however, remained high throughout the study period for our reed warblers, suggesting the activity level may be regulated by other information, such as for example the geomagnetic field (Ilieva et al. 2018, 2023). Our experiments further reveal a clear pattern of increased level of nocturnal migratory restlessness and a prolonged period of activity covering most of the dark period for both groups of reed warblers, as predicted for a long-distance nocturnal passerine migrant (Gwinner 1975, 1996; Berthold 1996; Newton 2008; cf. Ilieva et al. 2023).

The onset of nocturnal migratory activity of our reed warblers was tightly associated with the sunset period but contained some elevated pre-migratory activity before sunset. This pre-departure activity and following quiescence period expressed before the nocturnal restlessness have been reported in other captive studies of nocturnal passerine migrants (e.g. Watts et al. 2017; Müller et al. 2018; Åkesson et al. 2021) as well as in studies of free-flying migrants in the wild (e.g. Hebrard 1971; Åkesson et al. 1996; Bolshakov et al. 2007; Schofield et al. 2018). The pattern of activity and following rest in association with the sunset period have been shown to be very pronounced in nocturnally migrating European robins (*Erithacus rubecula*) kept in the same experimental set-up as our reed warblers (Bianco et al. 2019; Åkesson et al. 2021), suggesting a general function potentially associated with selecting a migratory direction for the upcoming flight. The time of this period overlaps with the availability of multiple cues for orientation (Åkesson et al. 1996), which opens for cue-compass calibrations (Muheim et al. 2006). The low activity could however also be associated with a need to sleep, as nocturnal migrants may suffer from sleep deficiency during migrations and use brief episodes for rest and REM sleep before initiating extensive flight periods (Rattenborg et al. 2004). Future studies need to reveal which factors play a role in explaining the behavioural shift and temporal variation in activity levels during this restricted period at sunset.

Our treatment reed warblers exposed to a delayed sunset, waited until the artificial lights went off before consistently showing nocturnal migratory restlessness behaviour, underlining the importance of external photic information associated with sunset as a trigger for the onset of migratory activity (Åkesson et al. 2021). A short light pulse has been shown to function as a trigger for increased activity in flying squirrels with immediate response the following days (DeCoursey 1960, 1961). An immediate response to a photic manipulation increasing daylength was found here for the reed warblers and in our previous studies of dunnocks and European robins (Åkesson et al. 2021), as well as for Northern wheatears (*Oenanthe oenanthe*) kept in cages (Müller et al. 2018). The immediate response is in line with the photic cue being used as a trigger to regulate diel activity patterns, including initiation of migration restlessness (Gwinner 1996, 2003; Helm et al. 2009).

In contrast to the pronounced and prolonged nocturnal activity period covering most of the dark part of the night, we found reduced daytime activity for both the control and treatment reed warblers demonstrating a substantial contrast in diel activity pattern between day and night for this species (Ilieva et al. 2023). This is in part unique for reed warblers as compared to other nocturnal passerine migrants studied in our laboratory where higher locomotor activities in association with feeding activity and searching were present at daytime and nocturnal restlessness was shorter each night or of overall lower activity (Bianco et al. 2019; Åkesson et al. 2021). The shorter nocturnal restlessness period observed for the European robin and common chiffchaff (*Phylloscpus collybita*) (Bianco et al. 2019; Åkesson et al. 2021) likely are associated with shorter anticipated migratory distances in autumn as compared to the reed warbler (Berthold 1973; Maggini and Bairlein 2010; Ilieva et al. 2023; this study), and the expressed activity patterns of the species could potentially be related to different optimal migration strategies related to their respective migration distance (Alerstam and Lindström 1990).

Studies of radio-tracked free-flying passerine migrants further show departure timing and length of nocturnal flight periods that may vary considerably between species and ecological situations (e.g. Åkesson et al. 1996, 2001, 2002; Bolshakov et al. 2007; Sjöberg et al. 2015, 2017; Müller et al. 2018), with birds initiating long enforced migration flight across barriers shortly after sunset, while flight passages over land may be initiated later relative to sunset (Müller et al. 2018). The prolonged nocturnal activity measured in our experiments may thus, be associated with the unique migration phenotype and the overall migratory strategy of reed warblers (Gwinner and Brandstätter 2001; Ilieva et al. 2023).

Reed warblers are known to put on lower amounts of fat at stopover during migration as compared to the closely related sedge warblers (*Acrocephalus schoenobaenus*) (e.g. Bibby and Green 1981; Stępniewska et al. 2018), which has been confirmed in a recent study, with the largest difference in food consumption of the two species during the initial part of a 13-day captive study period (Ilieva et al. 2023). By a low food consumption per day, reed warblers need to refuel longer and more frequently during migration and cannot perform as long continuous migration flights possibly lasting for several days as sedge warblers may do, especially in association with barrier crossings (Stępniewska et al. 2018; for review see, Ilieva et al. 2023). The two *Acrocephalus* species greatly differ in habitat choice, food preference and fuelling rates during migration, where sedge warblers are more dependent on aphids captured in reedbeds, enabling them to put on large fuel reserves fast (Stępniewska et al. 2018; Ilieva et al. 2023, and literature reviewed therein).

In conclusion, our experiments have revealed prolonged nocturnal restlessness for reed warblers recorded in cages, and an inherited flexibility in response to a modified diel cycle with respect to timing of activity and extent of fuelling. The experiments confirmed that the migration phenotype for nocturnal passerine migrants instantly respond to shifts in photic information (Åkesson et al. 2021), in line with light being used as a trigger to control circadian activity patterns (DeCoursey 1960, 1961). The increased diel period further affected the fuelling rate with an effect noted after one week of exposure, suggesting the endocrine system and physiological mechanisms involved in fuelling may need some time to respond to the changed diel cycle (Lindström 2003; cf. Sharma et al. 2021). We suggest that future studies should be directed to investigate what role different types of external photic and geomagnetic information play in regulating migration phenotype expression including activity and fuelling for birds adapted to migrate across different geographical regions, and whether interactions between external information and the endogenous program may contribute to the variation in migratory strategies observed in birds (Åkesson and Helm 2020). Ideally, controlled experiments in the laboratory should include detailed monitoring of relevant physiological processes (Sharma et al. 2021) and be compared with field-based methods to study circadian rhythms and migration in the wild (Dominioni et al. 2017; Guilford et al. 2011).

## Supplementary Information

Below is the link to the electronic supplementary material.

Supplementary file1 (PDF 128 KB)

## Data Availability

Activity data for the birds is provided as supplementary material.
