# Supplementary figures and images for: Effects of daylength manipulation on migratory activity and fuelling in a long-distance nocturnal songbird migrant
Source: J Comp Physiol A Neuroethol Sens Neural Behav Physiol. 2025 Oct 25;212(1):11–20. doi: 10.1007/s00359-025-01772-3 (PMC13038741; doi:10.1007/s00359-025-01772-3)

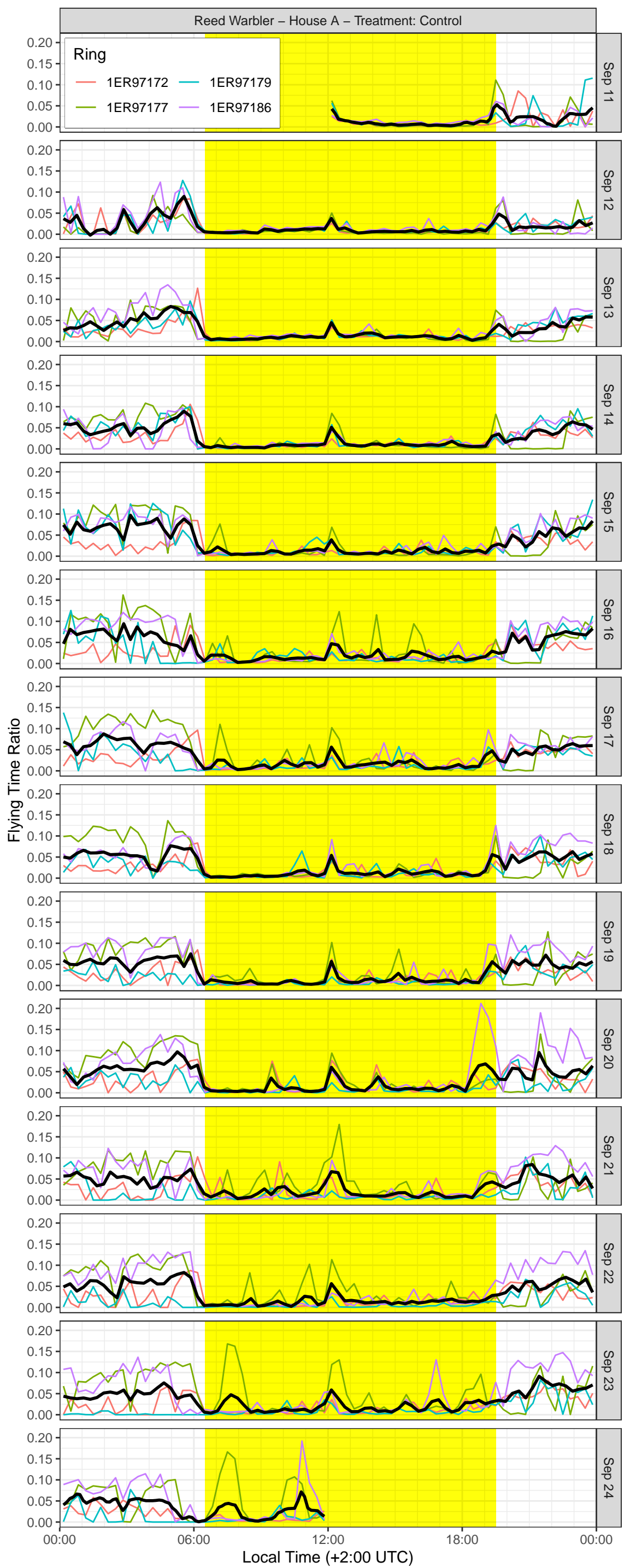

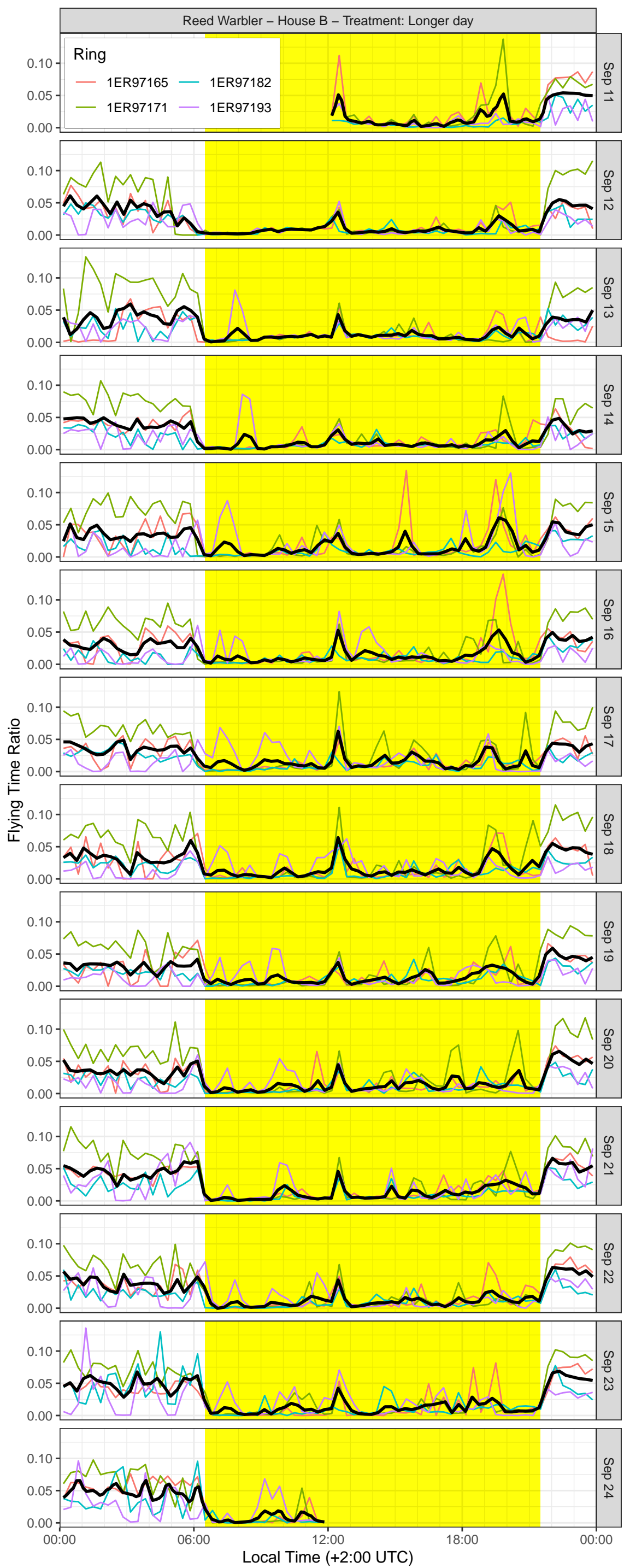

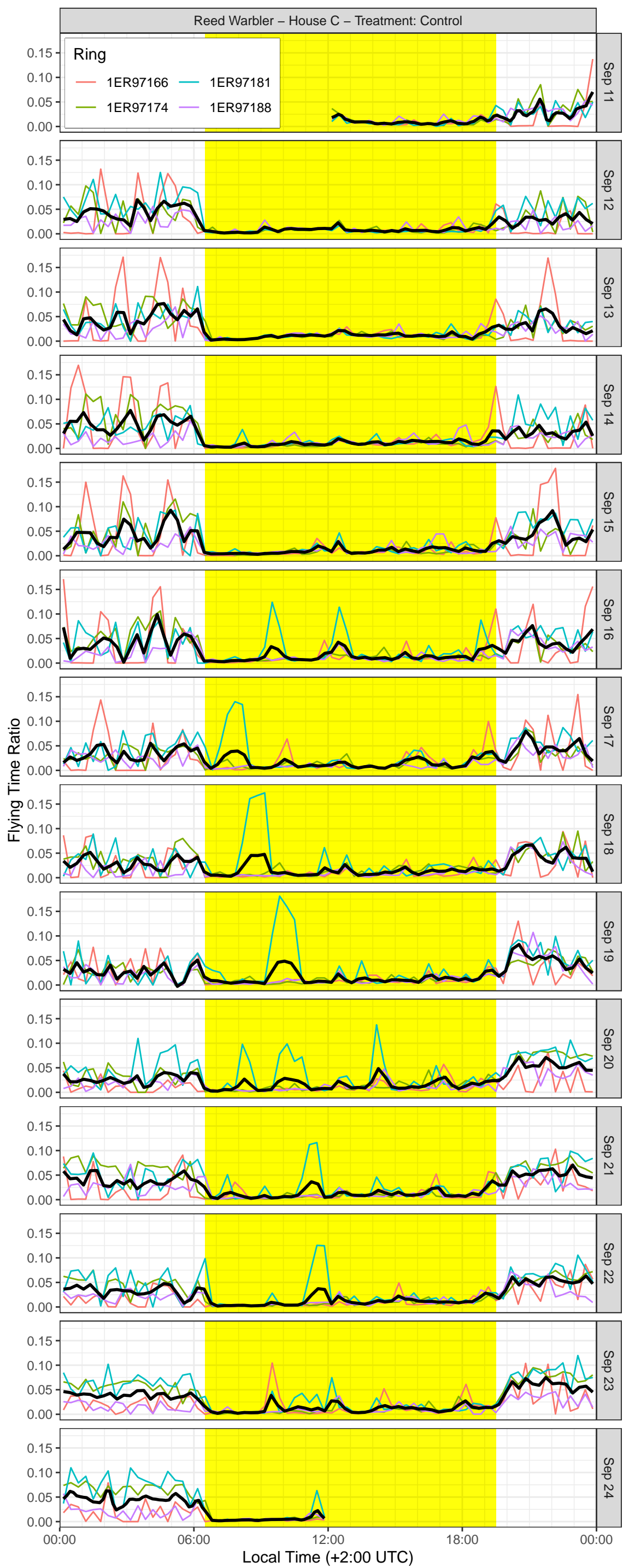

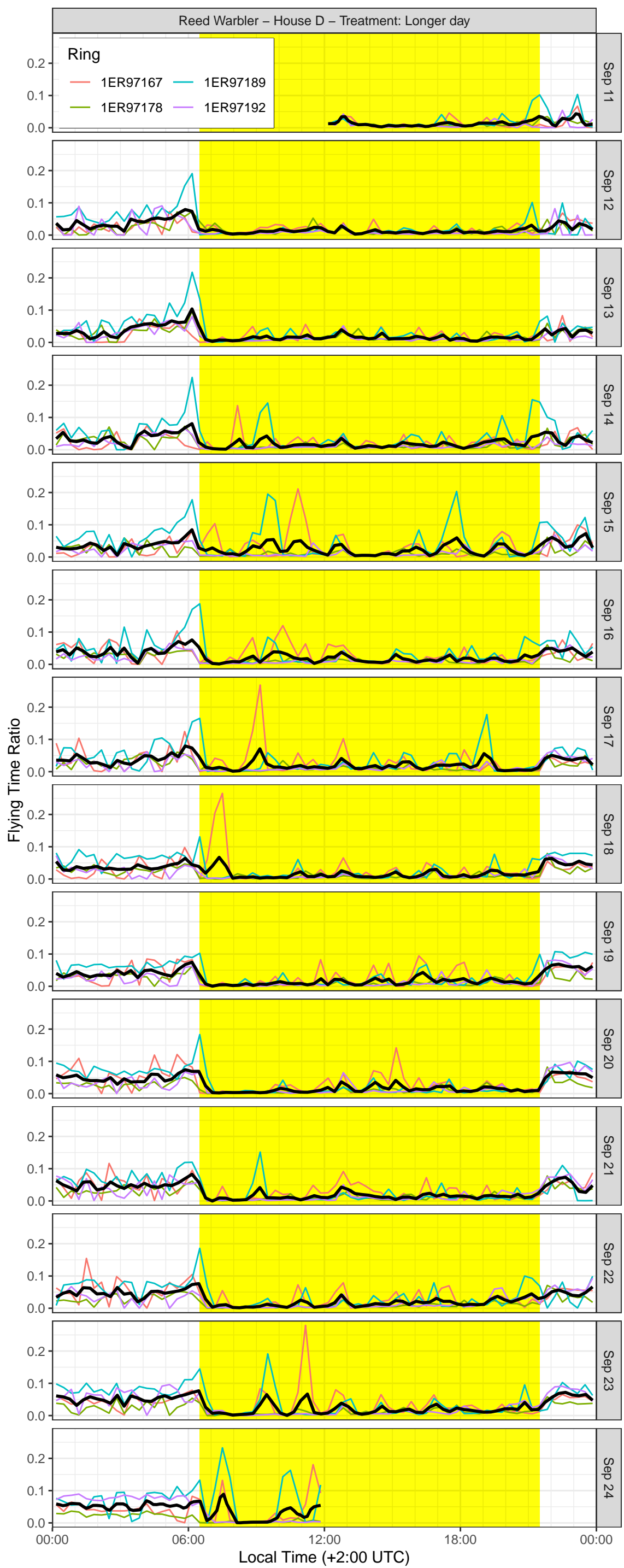

Supplement: Supplementary file 1 — Supplementary file1 (PDF 128 KB) [file 359_2025_1772_MOESM1_ESM.pdf]
